# Supplementary material for: Effects of Fluids on the Sublingual Microcirculation in Sepsis
Source: J Clin Med. 2022 Dec 8;11(24):7277. doi: 10.3390/jcm11247277 (PMC9786137; doi:10.3390/jcm11247277)
Supplement: Supplementary file 1 [file jcm-11-07277-s001.zip › jcm-2067870-supplementary.pdf]

Supplementary materials

Risk of bias tables methodology – from RevMan 5.4.

The following tables demonstrate the outcomes of risk of bias assessments done in RevMan 5.4 software. Randomised studies have been separated from the non-randomised observational studies as the categories and potential areas of bias are different. Non-randomised studies were assessed by each author under all domains using the ROBINS-I tool, available from Cochrane.

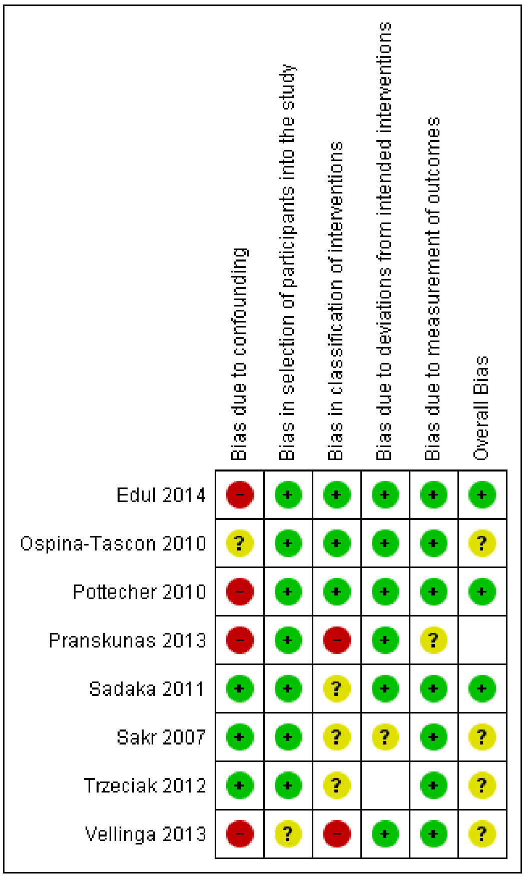

Figure S1(a) Risk of bias summary: review authors' Judgements about each risk of bias item for each included non-randomised study.

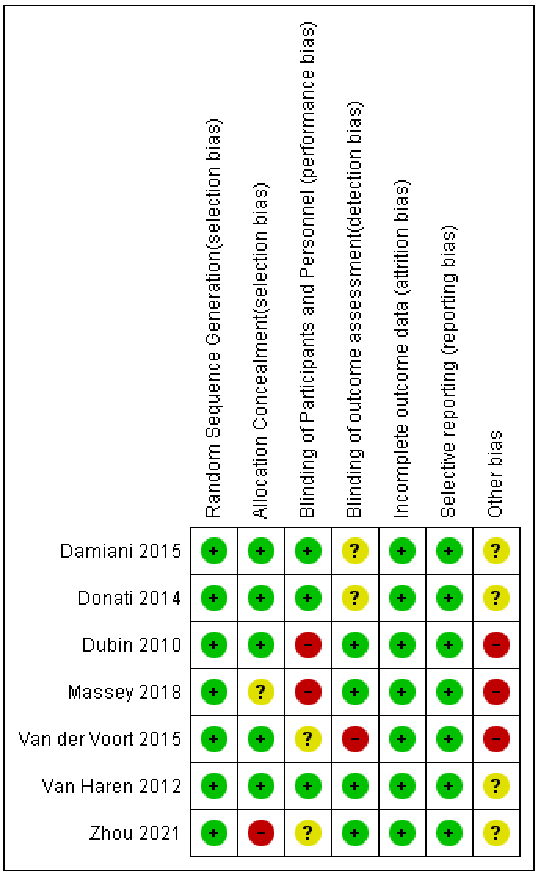

Figure S1(b) Risk of bias summary: review authors' judgements about each risk of bias item for each included randomised study

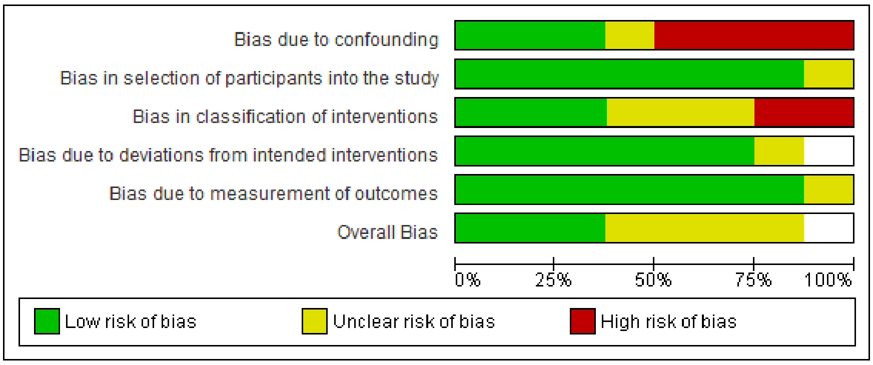

Figure S2(a) Risk of bias graph: review authors' judgements about each risk of bias

item presented as percentages across all included non-randomised studies.

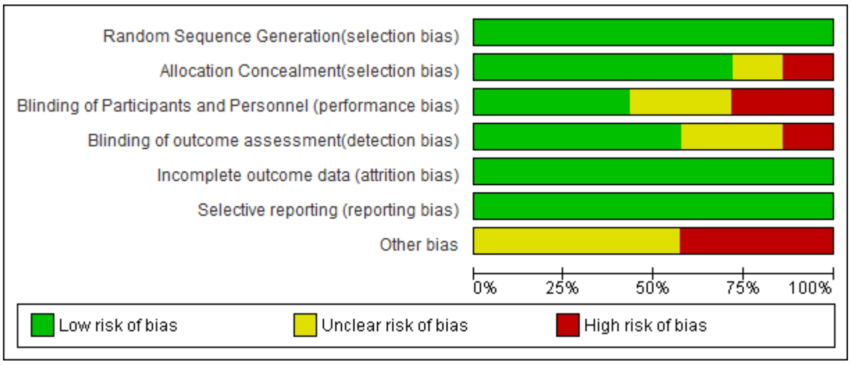

Figure S2(b) Risk of bias graph: review authors' judgements about each risk of bias item presented as percentages across all included randomised studies
